# A Simple Noninvasive Score Based on Routine Parameters can Predict Liver Cirrhosis in Patients With Chronic Hepatitis C

**DOI:** 10.5812/hepatmon.8352

**Published:** 2013-05-08

**Authors:** Ivan Gentile, Nicola Coppola, Giuseppe Pasquale, Raffaele Liuzzi, Maria D’Armiento, Maria Emma Di Lorenzo, Nicolina Capoluongo, Antonio Riccardo Buonomo, Evangelista Sagnelli, Filomena Morisco, Nicola Caporaso, Guglielmo Borgia

**Affiliations:** 1 Department of Clinical Medicine and Surgery, University of Naples Federico II, via S. Pansini, Naples, Italy; 2 Department of Public Medicine, Section of Infectious Disease, Second University of Naples, via L. Armanni, Naples, Italy; 3 Institute of Biostructure and Bioimaging, National Research Council (CNR), Naples, Italy; 4 Department of Advanced Biomedical Science, University of Naples Federico II, via S. Pansini, Naples, Italy

**Keywords:** Liver Cirrhosis, Fibrosis, Biopsy, Noninvasive Ventilation

## Abstract

**Background:**

Liver biopsy has remained the gold standard for the diagnosis of chronic hepatitis C; even though, it has a low but non-negligible rate of both false negative and complications. Several authors have proposed noninvasive tools to diagnose cirrhosis. But none of them showed complete concordance with liver biopsy.

**Objectives:**

To devise a score based on noninvasive routine parameters that discriminate between patients with a high risk, and those with a low risk of cirrhosis among patients with chronic hepatitis C without performing liver biopsy, and to compare this score with other ones using routine parameters devoted to this aim.

**Patients and Methods:**

We reviewed the charts of patients with chronic hepatitis C who performed a liver biopsy between 2000 and 2004. Multivariate analysis was used to identify independent predictors of cirrhosis. An independent group of patients with chronic hepatitis C admitted for a liver biopsy between 2007 and 2012 constituted the validation set.

**Results:**

We enrolled 249 patients who had complete laboratoristic data, and sufficient liver tissue for fibrosis staging. Age, AST, prothrombin activity, and platelets were identified as independent predictors of histological cirrhosis. We categorized these variables, and devised a novel score called CISCUN (Cirrhosis Score University of Naples), giving one point to each of the following predictors: age > 40 years; AST > 2 upper normal values; platelet count < 160.000/mmc; prothrombin activity < 100%. Cirrhosis rate was 2.9% for the 103 patients with a CISCUN = 0 or 1, 23.4% for the 124 patients with a CISCUN of 2 or 3, and 86.4% for the 22 patients with a CISCUN = 4. These results were confirmed in the independent validation group of 285 patients with similar characteristics.

**Conclusions:**

Patients with chronic hepatitis C and with a CISCUN ≤ 1 had a very low rate of cirrhosis while those with a CISCUN = 4 had a high risk of cirrhosis. Patients with CISCUN = 2 or 3 had an intermediate rate of cirrhosis, and therefore needed to perform a liver biopsy to receive a reliable diagnosis.

## 1. Background

Hepatitis C virus (HCV) is estimated to infect approximately 2.2–3.0% of the world’s population which corresponds to 130–170 million people (1). After penetrating in the host HCV gives rise to an acute infection which becomes chronic in about 70% of infected people (2, 3). About 25% of these patients would develop liver cirrhosis in about 20-30 years (4). About 4% per year of patients with cirrhosis would evolve toward a decompensated disease with an annual death rate between 15% and 30% (2). Finally about 1.6% of patients with cirrhosis develop hepatocellular carcinoma (HCC) yearly (2). Therefore the presence of cirrhosis is a main determinant of poor prognosis in patients with chronic HCV infection. Moreover, in the presence of cirrhosis, ultrasound screening for HCC, and upper endoscopy for varices detection are mandatory (5). Finally, the presence of cirrhosis influences decisions about antiviral treatment. Patients with cirrhosis have the most urgent need for treatment, but also a low response rate, and require a close monitoring during treatment (6). Liver biopsy is still considered the standard method for the assessment of liver cirrhosis, even though sampling errors and interpreter variability may reduce its diagnostic accuracy (7-10). Moreover liver biopsy is an invasive test, and therefore it has a small but not negligible rate of complications (0.3-0.8%), and death (0.01-0.3%) (11-14). Several groups have proposed noninvasive means to diagnose liver cirrhosis (15, 16). The most studied tools include the measurement of liver stiffness (17), and the use of a panel of selected blood tests, and a proprietary algorithm called Fibrotest (18) or a combination of both (19). Moreover several authors have proposed the use of nonroutinely available analytes (20-27) or routinely-available parameters to predict cirrhosis (6, 16, 28-40) or a combination of clinical and laboratoristic parameters (41). The accuracy of the noninvasive tools to predict cirrhosis in comparison to liver biopsy remains to be confirmed. The ideal noninvasive model that is easy-to-perform, accurately discriminate between patients with or without cirrhosis remains to be found.

## 2. Objectives

The aim of our study is to devise a score based on noninvasive routine parameters which can be helpful for physicians to identify both patients with a high risk and those with a low risk of having cirrhosis among a population of patients with chronic hepatitis C without performing liver biopsy (therefore that can safely avoid it), and to compare our score with other ones using routine parameters.

## 3. Patients and Methods

We reviewed the charts of all patients with chronic hepatitis C admitted for a liver biopsy to the Department of Public Medicine and Social Security – Section of Infectious Diseases (University of Naples “Federico II”, Italy), and to Department of Public Medicine, section of Infectious Disease (Second University of Naples) between 01.01.2000 to 31.12.2004. Liver biopsy was sampled through an 18G needle under ultrasound guidance. The liver specimens, in each case more than 2 cm in length, were fixed in 10% neutral buffered formalin, embedded in paraffin, and stained with hematoxylin and eosin, and with the Masson’s trichrome method. A sample of liver biopsies was examined by two pathologists who were unaware of the virological and clinical data, and who had a k index of 85.5%. Fibrosis was assessed according to the Ishak scoring system (42). Patients with stage 5 or 6 were considered having histological cirrhosis. Inclusion criteria were positivity for anti-HCV and HCV RNA in serum, no contraindication to liver biopsy. All the biopsies were performed at least 12 months after the completion of an eventual course of interferon therapy. Exclusion criteria were presence of other causes of liver disease; HBV or HIV coinfection, hepatocellular carcinoma, ascites, prior liver transplantation, insufficient liver tissue for staging of fibrosis, and incomplete data on blood counts or liver panel. Patients with a diagnosis of cirrhosis based on laboratory or ultrasound evaluation were excluded as well. Except for HCV genotype, only laboratory results performed within 1 month from the date of the liver biopsy were used. We recorded variables listed in Table 1. We also calculated the AST/ALT ratio. Splenomegaly was diagnosed when spleen length exceeded 12 cm at ultrasound examination (43). Alcohol consumption was defined as drinking more than 30 g per day of alcohol for at least 6 months. This was corroborated by the patient’s family. After the construction of the score, we validated it in an independent group of patients admitted for a liver biopsy to our department between 01.01.2007 and 30.06.2012. Inclusion and exclusion criteria were similar to those of the first group of patients.

**Table 1.** Demographic, Laboratory, Ultrasound, and Histological Features of the First Group of Patients (n = 249)

Items	Data
**Age, y, No. (Median)**	48 (35-54.5)
**Sex**	
Male, %	61.8
Female, %	38.2
**Alcohol consumption, %**	12
**Pervious Antiviral treatment, %**	13.2
**Splenomegaly at ultrasound, %**	23
**Iron, μg/mL, No. (Median)**	118 (85.5-148.5)
**Glucose, mg/dL, No. (Median)**	90 (80.25-101)
**Total Bilirubin, mg/dL, No. (Median)**	0.66 (0.50-0.87)
**AST^a^, U/L, No. (Median)**	62 (40.5-93)
**ALT^a^, U/L, No. (Median)**	106 (59-176)
**Alkaline phosphatase,U/L, No. (Median)**	149 (103.5-208)
**Albumin, g/dL, No. (Median)**	4.4 (4.2-4.6)
**Cholinesterase,U/L, No. (Median)**	10,754 (8,334-12613)
**White blood cells,cells/ μL, No. (Median)**	6,400 (5,370-7700)
**Red blood cells,cells/ μL, No. (Median)**	4,870,000 (4,625,000-5,190,000)
**Platelets, elements/μL, No. (Median)**	193,000 (159,000-234,000)
**Hemoglobin,g/dL, No. (Median)**	15.1 (14.2-15.9)
**Prothrombin activity, %**	99 (90-107)
**Alpha-fetoprotein,pg/mL, No. (Median)**	4 (2.3-8.1)
**Ferritin,ng/mL, No. (Median)**	162 (80-265.75)
**HCV^a^RNA, UI/mL, No. (Median)**	515,000 (200,000 – 850,000)
**Cirrhosis at liver biopsy, %**	20.5
**Staging at liver biopsy^b^, %**	
0	1.6
1	34.5
2	21.7
3	12.4
4	9.2
5	7.2
6	13.3

^a^Abbreviations: ALT, Alanine Aminotransferase; AST, Aspartate Aminotransferase; HCV, hepatitis C virus

^b^According to Ishak classification

### 3.1. Statistical Analysis

The Kolmogorov-Smirnov test was used to check quantitative variables for Gaussian distribution. In case of Gaussian distribution, data were reported as mean ± standard deviation (SD), while in case of non-Gaussian distribution they were reported as median and interquartile range (IQR). In case of Gaussian distribution, the Student’s t-test for unpaired variables was applied, while the Mann-Whitney U was used in case of non-Gaussian distribution. The chi-square test with Yates correction (or Fisher’s exact test where appropriate) was used for categorical variables. A P < 0.05 at two-sided test was considered statistically significant. Any independent variable statistically different in the two groups or with a P < = 0.2 at univariate analysis was included in binary logistic regression analysis using the forward conditional stepwise method. The cut-off values used for the stepwise method were: P = 0.05 for entry into the model, and P = 0.10 for its removal. We constructed a receiver operator characteristic (ROC) curve for each continuous variable. The curve shows the capacity of the variable to discriminate between patients with cirrhosis and those without it. The larger the area under the curve (AUC), the better the discriminating ability. An AUC above 0.7 is considered useful, and an AUC above 0.8 indicates excellent accuracy (44). All continuous variables were categorized to maximize easiness of use in clinical practice. Cutoffs were constructed by the means of ROC curve analysis based on the value that maximized the sum of sensitivity and specificity. AUCs of different ROC curves were compared according to the method of DeLong (45). We calculated specificity, sensitivity, positive predictive value, negative predictive value, diagnostic accuracy, positive likelihood ratio, negative likelihood ratio for our score, and compared these values with other scores available to the aim of predicting cirrhosis by noninvasive means. All statistical analyses were performed using the Statistical Package for the Social Sciences version 18.0 (SPSS Inc. Chicago, Ill).

## 4. Results

**Figure 1.**
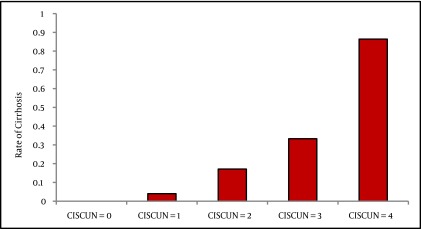
Rate of Cirrhosis in Patients With CISCUN 0-4 in the First Group of Patients (n = 249)

We enrolled 281 patients. We excluded 17 patients for insufficient liver tissue for staging of fibrosis, and 16 for incomplete biochemical or blood count data (one of them had also insufficient liver tissue). Therefore all analysis was performed on the remaining 249 patients. The main features of the 249 patients are shown in Table 1. In 51/249 patients (20.5%) histologic evaluation showed liver cirrhosis. Table 2 shows the demographic, laboratory, and ultrasound features of the 249 patients stratified according to the presence/absence of cirrhosis, and the results of univariate analysis. We put variables associated with presence of cirrhosis or with a P ≤ 0.2 at univariate analysis in a binary logistic regression analysis to identify independent predictors of cirrhosis. As shown in Table 3 higher age and AST, lower prothrombin activity and platelets were independent predictors of histological cirrhosis. We categorized these variables according to the ROC curves (see method section). The cutoff points generated were: age > 40 years; AST >2 upper normal values; platelet count < 160.000/mmc; prothrombin activity < 100%. We devised a score based on these predictors assigning 1 point per predictor. We called this novel score CISCUN (Cirrhosis Score University of Naples). Cirrhosis rate was 0% in the 28 patients with CISCUN = 0; 4% in the 75 patients with CISCUN = 1; 17.1% in the 76 patients with CISCUN = 2; 33.3% in the 48 patients with CISCUN = 3; and 86.4% in the 22 patients with CISCUN = 4 (see Figure 1). Therefore, as patients with CISCUN 0 or 1 had similar very low rate of cirrhosis, and those with CISCUN = 2 or 3 had similar intermediate risk compared to those with CISCUN = 4 which had a very different and high risk of cirrhosis, we decided to merge patients with CISCUN = 0 and 1, and consider them as patients at low risk, and to merge patients with CISCUN = 2 or 3 and consider them as patients in the “grey” area. Consequently, patients with a CISCUN of 0 or 1 had a very low risk of cirrhosis (2.9%) while patients with a score = 4 had a very high risk of cirrhosis (86.4%). Patients with a CISCUN of 2 or 3 showed an intermediate risk of cirrhosis (23.4%). We calculated the sensitivity (Se), specificity (Sp), positive predictive value (PPV), negative predictive value (NPV), diagnostic accuracy (DA), positive likelihood ratio (PLR), negative likelihood ratio (NLR) of the CISCUN using a cutoff of 1 (0-1 vs. 2-4), and using a cutoff of 3 (0-3 vs.4), and compared these with other scores predicting cirrhosis (see Table 4). We calculated AUC of the CISCUN and compared this value with other scores in the ability to predict cirrhosis (see Table 5). AUC of CISCUN was significantly higher than that of AST/AST ratio and Lok score. A trend toward higher AUC was observed for CISCUN compared to APRI and GUCI. In contrast AUC for CISCUN was not different from that of King’s college score. We validated CISCUN ability to discriminate between patients with and without cirrhosis in the independent set of patients with chronic hepatitis C. This group was composed of 285 patients admitted for a liver biopsy to our Departments between 01.01.2007 and 30.06.2012. Median age was 50 years (IQR: 39-58); median AST level was 52 U/L (IQR: 40-80); median Prothrombin activity was 101% (IQR: 94-108); median PLT levels was 217,000/µL (IQR: 178,000 – 279,500). Male represented 57.2% of subjects. Cirrhosis was identified in 27 (9.5%) cases. In these patients cirrhosis rate was 2.6% in the 39 patients with CISCUN = 0; 0.9% in the 117 patients with CISCUN = 1; 2.7% in the 73 patients with CISCUN = 2; 21.1% in the 38 patients with CISCUN = 3; and 83.3% in the 18 patients with CISCUN = 4. Therefore, even in this group it is confirmed that subjects with a CISCUN = 0 or 1 had a very low risk of cirrhosis (1.28%) while patients with a score = 4 had a very high risk of cirrhosis (83.3%). Patients with a CISCUN of 2 or 3 showed a non-negligible risk of cirrhosis (9%). Table 6 shows sensitivity, specificity, PPV, NPV, DA, PLR, and NLR of the CISCUN in the validation set.

**Table 2.** Demographic, Laboratory, Ultrasound Features of the First Group of Patients Stratified by Presence or Absence of Histological Cirrhosis (n = 249)^b^

	Patients without cirrhosis (n = 198)	Patients with cirrhosis (n = 51)	P value
**Age, y, No (Median)**	46 (33-54)	52 (45-56)	< 0.001
**Sex, %**			0.037
Male	58.6	74.5	
Female	41.4	25.5	
**Alcohol consumption, %**	13.4	17.5	0.500
**Pervious Antiviral treatment, %**	11.6	19.6	0.133
**Splenomegaly at ultrasound, %**	22	36.2	0.045
**Iron, µg/mL**	114 (83-143)	151 (112-183)	< 0.001
**Glucose, mg/** **dL**	88 (79-99)	93 (86-109)	0.002
**Total Bilirubin, mg/** **dL**	0.61 (0.48-0.80)	0.73 (0.59-1.04)	0.002
**AST^a^, U/L**	56 (39-83)	94 (64-119)	< 0.001
**ALT^a^, U/L**	93 (54-159)	139 (83-198)	0.001
**Alkaline phosphatase** **, U/L**	147 (95-205)	219.5 (130-296)	0.001
**Albumin, g/** **dL**	4.4 (4.2-4.6)	4.3 (4.1-4.5)	0.020
**Cholinesterase,U/L**	11,062 (8,799-12,996)	8,949 (6,892-11,270)	0.001
**White blood cells,cells/ μL**	6,450 (5,300-7,800)	6,400 (5,500-7,300)	0.541
**Red blood cells,cells/ μL**	4,860,000 (4,630,000-5,190,000)	4,910,000 (4,570,000-5,190,000)	0.765
**Platelets, elements/μL**	198,500 (171,750-245,000)	149,500 (118,000-188,000)	< 0.001
**Hemoglobin,g/dL**	15.0 (14.1-15.9)	15.4 (14.7-16.1)	0.044
**Prothrombin** ** activity,** **%**	101 (93-108)	92 (86-99)	< 0.001
**Alpha-fetoprotein,pg/mL**	3.3 (2.2-5.8)	8.1 (3.9-17.2)	< 0.001
**Ferritin, ng/mL**	137 (61-234)	195 (130-445)	0.037
**HCV^a^RNA, UI/mL**	511,000 (200,000–850,000)	698,000 (126,500–980,000)	0.541

^a^Abbreviations: ALT, Alanine Aminotransferase; AST, Aspartate Aminotransferase; HCV, hepatitis C virus

^b^Data were compared using χ ^2^ test for categorical variables and Mann-Whitney U test for quantitative variables

**Table 3.** Independent Predictors of Cirrhosis at Logistic Regression Analysis in the First Group of Patients (n = 249)

	Regression coefficient	Standard Error	Odds Ratio, (95% CI^a^)	P value
**Age, y**	0.059	0.019	1.061 (1.021-1.102)	0.002
**AST^a^, U/L**	0.04	0.02	1.004 (1.001-1.008)	0.046
**Prothrombin** ** activity,** **%**	-0.53	0.017	0.949 (0.919-0.980)	0.002
**Platelets,10^3^elements/μL**	-0.014	0.004	0.986 (0.978-0.994)	0.001

^a^Abbreviation: AST, Aspartate Aminotransferase; CI, Confidence Interval

**Table 4.** Sensitivity, Specificity, Positive Predictive Value, Negative Predictive Value, Diagnostic Accuracy, Positive Likelihood Ratio, Negative Likelihood Ratio for CISCUN, and For Other Noninvasive Scores Predicting Cirrhosis in the First Group of Patients (n = 249)

	Level	All Patients, No.	Patients. with cirrhosis, No. (%)	SE^a^, %	SP^a^, %	PPV^a^, %	NPV^a^, %	DA^a^, %	PLR^a^	NLR^a^
**CISCUN**	0-1	103	3 (2.9)	94.1	50.5	32.9	97.1	59.4	1.90	0.12
	2-4	146	48 (32.9)							
	0-3	227	32 (14.1)	37.3	98.5	86.4	85.9	85.9	24.59	0.64
	4	22	19 (86.4)							
**Lok** ** score ** **(6)**	≤ 0.2	99	6 (6.1)	88.2	47	30	93.9	55.4	1.66	0.25
	>0.2	150	45 (30)							
	≤ 0.5	222	36 (16.2)	29.4	93.9	55.6	83.8	80.7	4.85	0.75
	> 0.5	27	15 (55.6)							
**AST/ALT^a^ratio (39)**	< 1	227	46 (20.3)	9.8	91.4	22.7	79.7	74.7	1.14	0.99
	≥ 1	22	5 (22.7)							
**GUCI** **(33)**	≤ 1	129	7 (5.4)	86.3	61.6	36.7	94.6	66.7	2.25	0.22
	> 1	120	44 (36.7)							
**King’s college ** **(29)**	< 16.6	135	9 (6.7)	82.4	63.6	36.8	93.3	67.5	2.26	0.28
	≥ 16.6	114	42 (36.8)							
**APRI^a^ (40)**	≤ 1	151	12 (7.9)	76.5	70.2	39.8	92.1	71.5	2.57	0.34
	> 1	98	39 (39.8)							
	≤ 2	217	36 (16.6)	29.4	91.4	46.9	83.4	78.7	3.43	0.77
	> 2	32	15 (46.9)							
**PLT^a^ (35)**	≥ 150.000/mmc	203	25 (12.3)	51	89.9	56.5	87.7	81.9	5.05	0.55
	< 150.000/mmc	46	26 (56.5)							

^a^Abbreviations: SE, Sensitivity; SP, Specificity; PPV, Positive Predictive Value; NPV, Negative Predictive Value; DA, Diagnostic Accuracy; PLR, Positive Likelihood Ratio; NLR, Negative Likelihood Ratio; AST, Aspartate Aminotransferase; ALT, Alanine Aminotransferase; GUCI, Goteborg University Cirrhosis Index; APRI, AST to platelet ratio index; PLT, Platelet Count

**Table 5.** Comparison of CISCUN With Other Cirrhosis Scores

	AUC^a^	Standard Error	95% CI^a^	P value^b^
**CISCUN**	0.842	0.0300	0.790 to 0.885	
**APRI^a^**	0.793	0.0332	0.737 to 0.842	0.0604
**AST/ALT ratio^a^**	0.600	0.0405	0.536 to 0.662	< 0.0001
**GUCI^a^**	0.799	0.0328	0.743 to 0.847	0.0800
**King **	0.813	0.0315	0.758 to 0.860	0.1496
**L** **ok**	0.781	0.0368	0.725 to 0.831	0.0493

^a^Abbreviations: AUC, Area Under the Curve; CI, Confidence Interval; APRI, AST to platelet ratio index; AST, Aspartate Aminotransferase; ALT, Alanine Aminotransferase; GUCI, Goteborg University Cirrhosis Index

^b^Comparison with CISCUN

**Table 6.** Sensitivity, Specificity, Positive Predictive Value, Negative Predictive Value, Diagnostic accuracy, Positive Likelihood Ratio, Negative Likelihood Ratio for CISCUN in the Validation Set (n = 281)

	Level	All patients, No.	Patients with cirrhosis, No. (%)	SE^a^, %	SP^a^, %	PPV^a^, %	NPV^a^, %	DA^a^, %	PLR^a^	NLR^a^
**CISCUN**	0-1	156	2 (1.3)	92.6	59.7	19.4	98.7	62.8	2.30	0.12
	2-4	129	25 (19.4)							
	0-3	267	12 (4.5)	55.6	98.8	83.3	95.5	94.7	47.78	0.45
	4	18	15 (83.3)							

^a^Abbreviations: SE: Sensitivity; SP: Specificity; PPV, Positive Predictive Value; NPV, Negative Predictive Value; DA; Diagnostic Accuracy; PLR, Positive Likelihood Ratio; NLR, Negative Likelihood Ratio

## 5. Discussion

**Figure 2.**
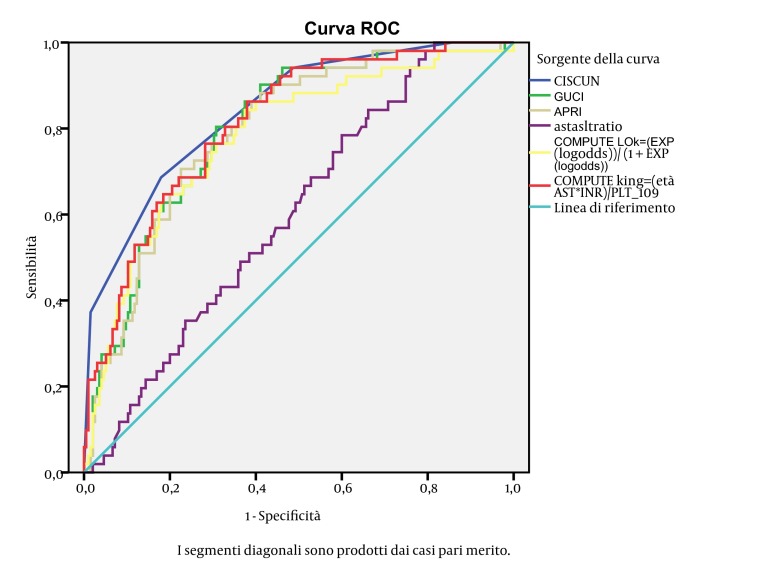
ROC Curves for the Scores Predicting Cirrhosis

In our study we identified 4 predictors of cirrhosis in a population of patients with chronic hepatitis C. These predictors were categorized to maximize the easiness of use in clinical practice. Patients with none or one of these predictors (CISCUN = 0 or 1) presented a very low risk of cirrhosis, while patients with all predictors (CISCUN = 4) had a high risk of cirrhosis. The patients in the “grey” zone (CISCUN 2 or 3) presented an intermediate risk of cirrhosis. As about a half of patients present a SCORE of 0, 1 or 4, liver biopsy may be avoided in about a half of patients with a low risk of misclassifying patients. We underline that the patients misclassified are 4.8% of patients with a score of 0, 1 or 4 and this Figure 2 is very low considering that liver biopsy itself has a rate of misclassification that reaches 11-20% in same studies (8, 46). These results were confirmed in an independent validation set of patients with HCV-related chronic liver disease. In this latter group more than 60% of liver biopsy (patients with CISCUN = 0, 1, or 4) may be avoided with an even lower risk of misclassification (2.9%). This is particularly interesting because the validation set showed a reduced rate of cirrhosis compared to the first group of patients. The diagnosis of cirrhosis remains a key element from both prognostic and therapeutic points of view. For example, the recent availability of protease inhibitors–containing combinations for patients infected with genotype 1, poses a series of problems. Costs and side effects of these drugs may represent a limitation to their use, considering that other and better tolerated drugs are in an advanced phase of clinical development. Decisions regarding immediate treatment or “wait and see” strategy cannot disregard a precise staging of the disease. The presence of cirrhosis is certainly a factor inducing an immediate treatment with the best options available, and liver biopsy still represents the gold standard to diagnose liver cirrhosis. However, liver biopsy is an invasive test associated with both complications and costs (11-14). Therefore a score able to diagnose cirrhosis by noninvasive means can be useful and cost-effective for the clinicians. Our score has several advantages over other ones devised for the same aim:

1) It includes parameters that are routinely available and unbiased in contrast with other studies that include analyses that are difficult-to-perform in clinical practice (20-27) or include subjective variables (41). Moreover the cost of the determination of our parameters is very low.

2) There is no need for mathematic formulae as in some other studies (6, 28-30, 32-34, 40). This represents a clear advantage for easiness which is a key element to make the score potentially widely used at the bedside

3) Our score showed a very high diagnostic performance in our cohort group of patients. In fact NPV (for CISCUN ≤ 1), and PPV (for CISCUN = 4) yielded the highest values among other scores devoted to cirrhosis prediction. It is noteworthy that the rate of false negative of CISCUN is very low (< 3%). This is a relevant point for a screening test.

Other approaches used to diagnose cirrhosis by noninvasive means include the measurement of liver stiffness by Fibroscan (17), and the employment of a proprietary algorithm on a panel of selected blood tests called Fibrotest (18). Both are useful predictors of cirrhosis. However, they are not universally available nor cost-saving tools compared to scores that use routine parameters. We underline that CISCUN includes only biologically plausible variables. In fact, age is associated with progression to cirrhosis (47). A low platelet count is associated with liver cirrhosis because of splenic sequestration due to portal hypertension. A reduced hepatic thrombopoietin production has also been claimed as a factor causing platelet decrease in patients with cirrhosis (48). It is noteworthy that platelet levels are included in almost all noninvasive scores predicting the presence of cirrhosis or esophageal varices (16, 49). AST levels are associated with liver cirrhosis as well. It is thought that this occurs because of a greater mitochondrial damage (and therefore to enhanced AST release) occurs in patients with cirrhosis. In our study AST/ALT ratio was not found to be associated with cirrhosis. Lastly prothrombin activity is directly related to hepatic synthetic function, and therefore worsens with progression of fibrosis and loss of hepatocytes (6). A potential limitation of our study (like all studies devoted to noninvasive prediction of cirrhosis) is that liver biopsy can provide additional information for physician such as quantify the degree of fibrosis, and show signs of overlapping conditions (e.g. NASH or iron overload).

In conclusion, we devised a novel score called CISCUN which is based on four predictors (Age > 40 years; AST > 2 upper normal values; platelet count < 160.000/mmc; Prothrombin activity < 100%), and assigns 1 point per predictor. CISCUN can identify patients with a very low rate of cirrhosis (CISCUN = 0 or 1), and patients with a high risk of having cirrhosis (CISCUN = 4) among subjects with chronic hepatitis C. Patients with a CISCUN = 2 or 3 (grey zone) present an intermediate risk of cirrhosis, and therefore should perform a liver biopsy to receive a reliable diagnosis.
